# Comparative Study of the Effectiveness of Double Priming Agents in Cervical Ripening and Induction of Labor in Nulliparas

**DOI:** 10.7759/cureus.90847

**Published:** 2025-08-23

**Authors:** Priyanka Peethambaran, Isha Seth, Aditya Vikram, Haritha K Madhusoodanan, Deepti Sharma

**Affiliations:** 1 Obstetrics and Gynaecology, Vydehi Institute of Medical Sciences and Research Centre, Bengaluru, IND; 2 Obstetrics and Gynaecology, Amrita Institute of Medical Sciences and Research Centre/Amrita Vishwa Vidyapeetham, Faridabad, IND; 3 Obstetrics and Gynaecology, Lifeline Multi Speciality Hospital, Adoor, IND

**Keywords:** cervical ripening, dual priming, foley catheter, induction of labor, misoprostol, nulliparous women, oxytocin, vaginal delivery rate

## Abstract

Background: Induction of labor is the stimulation of contractions before the spontaneous onset of labor with or without ruptured membranes. The labor induction in the presence of an unfavorable cervix is associated with an increased likelihood of prolonged labor and increased incidence of chorioamnionitis and caesarean section. Consequently, performing cervical ripening before conventional methods of induction has become standard practice. Combined techniques have been proposed to improve the success rate of labor induction and to enhance the progression of labor by leveraging the synergistic effects of both pharmacological and mechanical methods. In this comparative study, we examine the effectiveness of using a Foley catheter with misoprostol versus a Foley catheter with oxytocin infusion, specifically focusing on the induction-to-delivery interval in nulliparous women.

Method: Ours is a cross-sectional study conducted on pregnant patients booked for antenatal care at the obstetrics and gynecology (OBG) department of Amrita Institute of Medical Sciences, Kochi, Kerala, India, between November 2018 and August 2020. The study population comprised primigravidae who were admitted at term for induction of labor using double priming agents. A patient was then assigned to one of the two induction methods using dual priming agents practiced in our institution for the induction of labor in a primigravida with an unfavorable cervical score. A study proforma was designed to record all the observations pertaining to primary and secondary objectives of the study. The quantitative variables, such as induction-to-delivery time, were expressed as mean+/-standard deviation. The categorical variables were expressed as frequency and percentage. To test the statistical difference in the time from a Foley catheter placement to delivery between the two groups (Foley + oxytocin and Foley + misoprostol), an independent t-test will be applied. To test the statistical significance of the association between labor characteristics, the presence or absence of maternal morbidity, and neonatal outcomes between the two groups, the chi-square test was applied.

Results: We compared two methods of induction of labor with dual priming agents in nulliparous women. Our study did not find any difference in the induction-to-delivery interval between the two methods. However, there was a marginal advantage of two hours in a Foley catheter with the concomitant oxytocin group (17.03 + 6.51 vs. 19.32 + 6.6 hours), the difference was not statistically significant (p=0.70). The normal vaginal delivery rate was better in the Foley catheter with the Pitocin group (100% vs. 80.6% of patients), and the difference was statistically significant (p=0.04). Caesarean section, maternal, and neonatal outcomes were comparable with both methods, with no distinct advantage or disadvantage of one over the other.

Conclusion: Over the past several decades, the rate of labor induction has risen significantly, a trend that is unlikely to reverse in light of increasing maternal morbidities. In response, novel strategies are needed to meet the growing demands on labor and delivery units. Combination methods offer a promising approach for expediting labor without adding risks for either the mother or newborn.

## Introduction

Over the past several years, obstetricians have shown great interest in the process of parturition, prompting extensive research on maternal well-being and the optimal timing of birth. This ongoing interest has led to the development of numerous strategies to initiate labor. Induction of labor is defined as the stimulation of uterine contractions before their spontaneous onset, with or without ruptured membranes [1].

Over recent decades, the incidence of labor induction has continued to rise. In some developed nations, as many as one in four term deliveries result from induction. Additionally, a World Health Organization (WHO) survey on maternal and perinatal health conducted in 24 countries revealed that 9.6% of deliveries were induced. Notably, Asian and Latin American countries recorded higher rates, such as Sri Lanka (35.5%), in contrast to some African nations such as Niger (1.4%) [2].

Induction of labor is generally indicated when the potential benefits for the mother or fetus outweigh the advantages of continuing the pregnancy. The most common indication is post-term pregnancy, which has helped reduce perinatal mortality. Other frequent reasons for induction include premature rupture of membranes, gestational hypertension, oligohydramnios, non-reassuring fetal status, and various maternal medical conditions [3]. Despite its benefits, labor induction carries a two- to threefold increased risk of cesarean delivery, particularly among nulliparous women [4]. Additional complications associated with induction include postpartum hemorrhage due to uterine atony, chorioamnionitis, and uterine rupture.

Various factors can increase the likelihood of a successful labor induction, including a favorable cervix, multiparity, ruptured membranes, lower body mass index (BMI), taller maternal stature, and lower estimated fetal weight [5]. The Bishop score remains the most commonly used tool for cervical assessment, evaluating the station of the presenting part alongside four cervical characteristics: dilation, effacement, consistency, and position. Of these, cervical dilation is generally considered the most important. Although there is no universal cutoff for differentiating favorable versus unfavorable scores, higher Bishop scores correlate with a greater chance of vaginal delivery, while lower scores are associated with an increased risk of cesarean delivery.

In practice, many obstetricians treat a Bishop score of 6 or more as favorable and 3 or less as unfavorable, with scores of 4 or 5 falling into a grey zone. When the cervix is deemed unfavorable, cervical ripening is recommended [6]. Prostaglandins are commonly used to improve the Bishop score prior to administering oxytocin, whereas oxytocin may be used directly if the cervix is already favorable [7].

Induction of labor may be achieved through pharmacological, mechanical, or combined methods. Ideally, any agent or technique used should replicate the natural progression of labor while avoiding excessive uterine hyperstimulation. In cases of an unfavorable cervix, common approaches include the intravaginal use of misoprostol, transcervical insertion of a Foley’s catheter, or application of prostaglandin gel to enhance cervical parameters. Conversely, if the cervix is deemed favorable, oxytocin infusion is generally preferred. Misoprostol has garnered increasing attention as a pharmacological agent for labor induction; however, considerable debate persists regarding the optimal dosage, route, and dosing interval [8].

Combined methods have been proposed to increase the likelihood of a successful induction and to accelerate labor progression through the synergistic effects of pharmacological and mechanical techniques [9]. Accordingly, our study provides a comparative analysis of two combined methods: a Foley catheter with misoprostol versus a Foley catheter with an oxytocin infusion, focusing on their impact on induction-to-delivery intervals in nulliparous women.

Despite ongoing research, the pursuit of an ideal labor induction combination remains unresolved. Efforts continue to enhance induction success rates by utilizing various methods either simultaneously or sequentially, adjusting drug combinations and dosages to decrease cesarean delivery rates, shorten the induction-to-delivery interval, and minimize both fetal and maternal complications. While numerous studies have compared different doses of misoprostol, Foley catheter induction against a Foley catheter plus oxytocin, or a Foley catheter plus misoprostol, few have examined dual agents used in combination. Our study specifically addresses this gap by comparing these dual-agent protocols, a topic that has not been previously investigated.

## Materials and methods

This cross-sectional study was conducted on pregnant individuals receiving antenatal care at the Department of Obstetrics and Gynecology, Amrita Institute of Medical Sciences, Kochi, Kerala, India, between November 2018 and August 2020. The study population comprised nulliparous women admitted at term for labor induction using dual priming agents.

The study included nulliparous women aged between 18 and 40 years who had a singleton gestation in cephalic presentation without fetal anomalies. Participants were admitted for induction of labor between 28 and 40 weeks of gestation and had a Bishop score of less than 6 at the time of admission. Women were excluded if they had multiple gestations, non-vertex fetal presentation, a known latex allergy, fetal anomalies, vaginal bleeding, or premature rupture of membranes.

All eligible patients were counseled and invited to participate in the study at the time of hospital admission. On the day of induction, their eligibility was reconfirmed, and a pelvic examination was performed to calculate the modified Bishop’s score prior to induction. After obtaining informed consent, a 16 Fr latex Foley catheter was inserted through the internal os under sterile conditions and then inflated with 60 mL of saline. Each patient was assigned to one of two induction methods (dual priming agents) routinely used in the institution for nulliparous women with unfavorable cervical scores.

A Foley catheter was removed 12 hours after insertion if it had not already been spontaneously expelled. Bishop’s score was recalculated at the time of a Foley catheter expulsion or removal. Subsequent management of labor followed the standard protocol of the labor ward.

A structured proforma was used to capture all observations relevant to the study’s primary and secondary objectives. The senior postgraduate on duty in the labor room recorded the induction-to-delivery interval and other labor characteristics before the patient was transferred to the postnatal ward. Maternal complications and neonatal outcomes were extracted from the patient’s medical records prior to hospital discharge.

Patients were continuously enrolled until the required sample size was reached.

Sample size

Sample size was calculated on the basis of the mean time (Foleys with PGE1-14 hours in Osoti et al.'s [10] study and Foley’s with Pitocin 20 hours in Schoen et al.'s [11] study) from Foley’s catheter placement to delivery at 24 hours or less, as observed in the previous two publications using dual priming agents.

Statistical analysis

Statistical analysis was performed using Statistical Product and Service Solutions (SPSS, version 20; IBM SPSS Statistics for Windows, Armonk, NY) software. Categorical variables were expressed using frequency and percentage. Numerical variables were presented using mean and standard deviation. An independent sample t-test was used to study the statistical significance of the comparison in the time from Foley’s catheter placement to delivery between the two groups (Foley's + oxytocin and Foley's + misoprostol). A chi-square test was used to study the statistical significance of the association of medical comorbidities, indication for induction, and delivery within 24 hours between both groups. To test the statistical significance of the association of vaginal delivery within 24 hours between the two groups, the chi-square test with continuity correction was used. Multivariate analysis was done to identify the independent predictors of induction outcomes. A p value of <0.05 was considered to be statistically significant.

## Results

Of the 106 women initially approached for this study at Amrita Institute of Medical Sciences between November 2018 and August 2020, a total of 92 participants were enrolled in this cross-sectional research. Fourteen patients were excluded because they presented in active labor (n=10) or had pre-labor rupture of membranes (n=4). In an additional two patients, a Foley catheter could not be inserted due to a markedly posterior cervix and maternal obesity. Ultimately, 90 participants completed the study with complete data.

The mean patient age, BMI, and gestational age (in weeks) at induction were computed for both groups. Although there were slight disparities in these variables - statistically significant differences were noted - participants in the Foley with the Pitocin group were younger (24.25 ± 2.60 years vs. 25.96 ± 4.08 years) and had a lower BMI (21.9 ± 1.97 kg/m² vs. 23.2 ± 3.37 kg/m²) compared to those in the Foley with PGE1 group. Conversely, mean gestational age at the time of induction was higher in the Foley with Pitocin group (38.9 ± 0.65 vs. 38.5 ± 1.05 weeks). Both groups were comparable in terms of indications for induction and associated medical comorbidities. Elective induction at term was the predominant reason for induction in both cohorts. Among participants in the Foley with Pitocin group, 27.5% had associated medical conditions compared to 18% in the Foley with PGE1 group, with gestational diabetes mellitus being the most common comorbidity across both groups. The most common indication for induction in both groups was planned induction at term (88% in the Foley with PGE1 group and 90% in the Foley with Pitocin group). The difference in these clinical variables between the two groups was not statistically significant (p>0.05) (Table 1).

**Table 1:** Distribution of the study population based on clinical characteristics Values are presented as mean ± SD or n (%). An independent t-test was used for continuous variables and a chi-square test for categorical variables. A p-value < 0.05 was considered statistically significant.

Characteristic	Foley + PGE1 (n=50)	Foley + Pitocin (n=40)	p-value
	Mean ± SD	Mean ± SD	
Age	25.96 + 4.08	24.25 + 2.60	0.01
BMI at the time of delivery (kg/m^2^)	23.2 + 3.37	21.9 +1.97	0.02
Gestational age (in weeks)	38.5 + 1.05	38.9 + 0.65	0.04
Medical Comorbidities			
Yes	9 (18%)	11 (27.5%)	0.281
No	41 (82%)	29 (72.5%)	
Indication for Induction			
Term	44 (88%)	36 (90%)	1.000
Others	6 (12%)	4 (10%)	

Induction-to-delivery interval

The mean time from induction to delivery was shorter by approximately two hours in the Foley with Pitocin group (17.03 ± 6.51 hours vs. 19.32 ± 6.6 hours); however, the difference was not statistically significant (p=0.70). Notably, 82.5% of patients in the Foley with Pitocin group delivered within 24 hours compared to 72% in the Foley with PGE1 group. While 28% of patients in the Foley with PGE1 group took longer than 24 hours to deliver, only 17.5% in the Foley with Pitocin group crossed this threshold. The statistical difference for both variables was not significant (p=0.24).

When evaluating only normal vaginal deliveries within 24 hours, a significant difference emerged between the two groups. All women (100%) in the Foley with Pitocin group achieved normal vaginal delivery, as opposed to 80.6% in the Foley with PGE1 group (p=0.04) (Table 2).

**Table 2:** Distribution of the study population in both the groups based on induction outcomes Values are presented as mean ± SD or n (%). The independent t-test was used for continuous variables, and the chi-square test for categorical variables. A p-value < 0.05 was considered statistically significant.

Induction outcomes	Foley + PGE1 (n=50)	Foley + pitocin (n=40)	p-value
	Mean ± SD	Mean ± SD	
Total time to delivery (in hours)	19.32 + 6.6	17.03 + 6.51	0.70
Delivery in 24 hours			
Yes	36 (72%)	33 (82.5%)	0.242
No	14 (28%)	7 (17.5%)	
Normal vaginal delivery in 24 hours			
Yes	25 (80.6%)	27 (100%)	0.04
No	6 (19.4%)	0	

Multivariate analysis

Because the two groups differed in age, BMI, and gestational age at induction (Table 1), a multivariate analysis was performed. After adjusting for these variables in relation to the outcome of “delivery within 24 hours,” the Foley with Pitocin group did not demonstrate a statistically significant independent effect for achieving delivery within 24 hours. In the univariate analysis examining normal vaginal delivery within 24 hours, a significant difference was observed. However, because there were no cases of women in the Foley with Pitocin group who had a normal vaginal delivery beyond 24 hours, multivariate analysis controlling for age and BMI could not be performed. A larger sample size might be needed in future research to assess the effect of the Foley with Pitocin on normal vaginal delivery within 24 hours (Table 3).

**Table 3:** Multivariate analysis with respect to delivery within 24 hours Multivariate logistic regression analysis was performed. A p-value < 0.05 was considered statistically significant.

Variables	B	Wald	p-value	OR	95% CI for EXP(B)	
					Lower	Upper
Age (≤25)	0.149	0.074	0.786	1.161	0.396	3.405
GA (≤38)	0.468	0.458	0.498	1.596	0.412	6.186
BMI	0.108	1.386	0.239	1.114	0.931	1.335
Medical comorbidities (Yes)	-0.293	0.171	0.679	0.746	0.186	2.993
Indication for Induction (Term)	0.539	0.323	0.570	1.715	0.267	11.029
Group (Foley - PGE1)	-0.520	0.859	0.354	0.595	0.198	1.786

Labor progress and bishop score changes

The time required for spontaneous expulsion of the Foley catheter after initial placement was similar in both groups. The change in Bishop’s score at the time of Foley’s catheter expulsion, compared to the pre-induction score, appeared more favorable in the Foleys with Pitocin group (10.07 ± 2.88 vs. 7.3 ± 3.31). Additionally, the time from Foley’s catheter expulsion to full cervical dilation was shorter (6.10 ± 3.25 vs. 15.9 ± 5.89 hours) in the Foley with Pitocin group, though this difference was not statistically significant. Because of a more pronounced Bishop’s score improvement in this group, these patients underwent artificial rupture of membranes (ARM) earlier, which contributed to reducing the time taken to reach full dilation. In contrast, participants in the Foley with PGE1 group did not consistently achieve Bishop’s score criteria for ARM as an augmentation method.

When comparing the proportion of women who delivered within 12 hours, the Foley with Pitocin group showed a slight advantage (32% vs. 24%), although this difference did not reach statistical significance (p=0.37). Regarding analgesic requirements, while the Foley catheter remained in situ, the two groups were similar, with about 40% of patients in each group requesting pain relief during the latent phase.

Mode of delivery and maternal outcomes

Non-progression of labor was the most common indication for cesarean section in both groups, followed by fetal distress. Approximately 5% of patients in the Foley with Pitocin group failed to enter the active phase of labor, as did 2% of those in the Foley with PGE1 group. No significant differences in maternal complications were noted between groups (Table 4).

**Table 4:** Comparison of labor characteristics between the two groups Values are presented as mean ± SD or n (%). The independent t-test was used for continuous variables, and the chi-square test for categorical variables. A p-value < 0.05 was considered statistically significant.

Induction outcomes	Foley + PGE1 (n=50)	Foley + Pitocin (n=40)	p-value
	Mean ± SD (in hours)	Mean ± SD (in hours)	
Time to Foley expulsion	6.9 + 3.4	6.63 + 3.33	0.137
Time to full dilatation	15.91 + 5.89	6.10 + 3.24	0.137
Change in Bishop’s score by 12 hours	7.3 ± 3.31	10.07± 2.88	0.13
Delivery in 12 Hours			
Yes	12 (24%)	13 (32%)	0.37
No	38 (76%)	27 (67%)	
Regional Analgesia During a Foley			
Used	18 (36%)	17 (42.5%)	0.530
Not used	32 (64%)	23 (57.5%)	
Indication for Caesarean Delivery			
Non-progression of labour	10 (20%)	4 (10%)	
Fetal distress	6 (12%)	4 (10%)	
Occipito-posterior	1 (2%)	0	
Failed induction	1 (2%)	2 (5%)	
Cephalopelvic disproportion (CPD)	1 (2%)	0	

Among women who ultimately required cesarean section, the mean age was 26.63 ± 3.22 years versus 25.07 ± 3.74 years among those delivering by other means - a difference that was not statistically significant (p=0.460). However, the mean BMI was significantly higher in the cesarean group (23.7 ± 3.20) compared to the non-cesarean group (22.1 ± 2.64; p=0.019), suggesting that elevated BMI may be associated with an increased likelihood of cesarean delivery (Table 5).

**Table 5:** Association of age and BMI with the mode of delivery Values are presented as mean ± SD. The independent t-test was used. A p-value < 0.05 was considered statistically significant.

	Caesarean (n=27)	Others (n=63)	p-value
	Mean ± SD	Mean ± SD	
Age	26.63 ± 3.22	25.07 ± 3.744	0.460
BMI	23.7 ± 3.20	22.1 ± 0.641	0.019

Both induction methods proved safe in terms of maternal complications. There were no cases of uterine rupture, ICU admissions, or maternal death. Uterine hyperstimulation was observed in two patients in each group, effectively managed by reducing the oxytocin infusion rate in the Foley with Pitocin group. Two patients in the Foley with PGE1 group experienced postpartum hemorrhage and intrapartum fever. No statistically significant difference was noted between the two groups for maternal complications (p=0.3).

Neonatal outcomes

Respiratory distress was the most common neonatal complication, occurring in 17% of infants in the Foley with PGE1 group and 5% in the Foley with Pitocin group, with no statistically significant difference between groups (p=0.579). In the Foley with Pitocin group, both infants who developed respiratory distress also had meconium-stained liquor and required NICU observation. Approximately 10% of neonates in the Foley with PGE1 group needed oxygen support in the NICU; however, none required mechanical ventilation or prolonged NICU stays.

Mean neonatal birth weight (2.91 ± 0.35 kg vs. 2.96 ± 0.36 kg) and cord pH values (7.38 ± 0.08 vs. 7.39 ± 0.04) were comparable in the two groups. However, neonates in the Foley with Pitocin group had significantly better FIVE-minute Apgar scores (8.62 ± 0.53 vs. 8.85 ± 0.36; p=0.01) (Table 6).

**Table 6:** Distribution of maternal and neonatal adverse outcomes between the two groups Values are presented as n (%). The chi-square or Fisher’s exact test was used for categorical variables. A p-value < 0.05 was considered statistically significant.

Variables	Foley + PGE1 (n=50)	Foley + Pitocin (n=40)	p-value
	No. (%)	No. (%)	
Maternal complications			
Present	4 (8%)	2 (5%)	0.321
Absent	46 (92%)	38 (95%)	
Neonatal complications			0.579
Present	7 (17%)	2 (5%)	
Absent	43 (83%)	38 (95%)	
NICU Admission			0.379
Yes	5 (10%)	2 (5%)	
No	45 (90%)	38 (95%)	
Liquor			0.110
Yes	0	2 (5%)	
No	50 (100%)	38 (95%)	

Overall, these findings suggest that both the Foley catheter with Pitocin and the Foley catheter with PGE1 are viable options for labor induction in nulliparous women with unfavorable cervices, each demonstrating comparable maternal safety and neonatal outcomes, with some potential advantages observed in the Foley with Pitocin group.

## Discussion

Both mechanical and pharmacological agents are used for labor induction, and research suggests that using each method individually can reduce the incidence of cesarean delivery [12]. Combining mechanical and pharmacological methods may therefore have a synergistic effect, potentially shortening labor and decreasing the induction-to-delivery interval. Previous investigations have reported conflicting results: while some show promise in reducing labor duration and cesarean risk, others do not.

In our study, the use of a Foley catheter concurrently with Pitocin shortened the induction-to-delivery interval by almost two hours compared to the combined Foley catheter and misoprostol approach, although this difference did not reach statistical significance (p=0.7). Notably, both groups in our study received dual-agent regimens; thus, our findings cannot be directly compared to studies contrasting single-agent versus combination protocols.

When examining dual-agent approaches separately, our mean induction-to-delivery interval of 17.03 hours in the Foley catheter plus Pitocin group compares favorably with the 20.9-hour interval reported by Schoen et al. [11], although it is slightly longer than the 15.9-hour interval reported by Conolly et al. [13]. Differences in study populations, protocols, and clinical settings may account for these variations.

Similarly, the mean induction-to-delivery interval in our Foley catheter with concurrent misoprostol group was 19.32 hours, notably longer than the 14.5 hours reported by Levins et al. [9]. In their study, four groups were compared: a Foley alone, misoprostol alone, a Foley combined with misoprostol, and a Foley combined with Pitocin. In contrast, Osoti et al. [10]. documented a mean induction-to-delivery interval of 14.1 hours for a combined Foley plus misoprostol regimen, which also outperformed our finding of 19.3 hours.

In our investigation, we observed a significant difference (p=0.04) in the proportion of patients achieving vaginal delivery between the two study groups: 100% of those in the concurrent Foley with Pitocin group delivered vaginally, compared to 80.6% in the Foley with PGE1 group. Moreover, the induction-to-full-dilation interval in the concurrent Foley with Pitocin group was nine hours shorter than in the Foley with PGE1 group.

Conversely, Pettkar et al. [14]. noted no difference in the proportion of patients delivering vaginally within 24 hours between their study groups (48% in the concurrent Foley group vs. 46% in the Foley-alone group). However, the groups in that study differed in terms of parity. By contrast, our research focused exclusively on nulliparous women, allowing for a more direct evaluation of the efficacy of induction methods in this specific population.

Osoti et al. [10] found no statistically significant increase in the proportion of women achieving vaginal delivery within 24 hours when using a combined method (80% in the Foley plus misoprostol group) compared to those receiving misoprostol alone (84.4%). In contrast, Schoen et al. [11] observed that 92% of women in the Foley with concurrent oxytocin group delivered vaginally within 24 hours, compared to only 71% in those who received a Foley catheter alone, followed by oxytocin (p=0.004).

In our study, the total time to reach full dilation was nine hours shorter in the concurrent Foley with Pitocin group than in the Foley with misoprostol group, although this was not statistically significant (p=0.137). These results align with the findings of Schoen et al. [11] who reported a significant three-hour reduction in mean time to full dilation among women who received concurrent Foley with oxytocin (13.1 hours vs. 16.5 hours in the Foley-then-oxytocin group; p=0.004).

Ultimately, the success of labor induction hinges on achieving a safe vaginal delivery with minimal complications and without increasing the cesarean delivery rate.

In our study, the overall proportion of women who required cesarean deliveries was similar between the two groups (34% in the Foley with PGE1 group vs. 30% in the concurrent Foley with Pitocin), with non-progression of labor being the most frequent indication for cesarean delivery. These results are consistent with the findings of Conolly et al. [13]. In their study, the most common indication for cesarean delivery was a non-reassuring fetal heart rate pattern.

Schoen et al. [11] similarly reported no significant difference in cesarean delivery rates between their two study arms (13% vs. 16%). For nulliparous women in their cohort, arrest of dilation was the primary indication for cesarean in the Foley with oxytocin group (53% vs. 23%; p=0.005). Osoti et al. [10] likewise found no significant difference in cesarean delivery rates (20% in the combined Foley with misoprostol group vs. 15% in the vaginal with misoprostol group). Notably, in our study, patients who underwent cesarean delivery tended to have a higher BMI (23.7 kg/m² in the cesarean group vs. 22.1 kg/m² in the non-cesarean group; p=0.019). By comparison, Conolly et al. [13] identified advanced maternal age (>35 years) as an important factor associated with cesarean delivery (65% vs. 37% in the non-cesarean group; p=0.005), whereas obesity did not significantly influence the mode of delivery in their population.

Pettkar et al. [14] additionally noted an increased need for analgesia among patients who received concurrent Foley with Pitocin (23% vs. 9%; p=0.01; RR: 2.60, 95% CI: 1.21-5.56). This finding was not replicated in our study or in Schoen et al. [11].

Regarding maternal and neonatal outcomes, we observed no statistically significant differences between the two dual-agent protocols, echoing the observations from other studies. Both methods proved equally safe from maternal and neonatal standpoints, with no major complications such as uterine rupture, severe maternal morbidity, or poor neonatal outcomes attributable to either regimen.

Although our data showed no significant difference in the induction-to-delivery interval between these two dual-priming methods, our sample size of 90 patients was relatively small. Future prospective trials with larger and more diverse populations - including multiparous women, those with pre-labor rupture of membranes, and multifetal gestations - would provide greater insight into the optimal approach for combining these agents in labor induction.

Strengths of the study

A key strength of this research is that it is the first to directly compare dual priming agent regimens in nulliparous women. Previous studies on labor induction have primarily examined single agents (e.g., misoprostol alone, Foley with misoprostol, Pitocin alone, or Foley with Pitocin) or focused on different dosage protocols of a single pharmacological agent. By using strict inclusion and exclusion criteria, our study provides a more targeted assessment of the efficacy and safety of these dual-method approaches in a well-defined population.

Limitations of the study

One notable limitation is the demographic disparity between the two groups, particularly regarding mean age and BMI. Because this was a cross-sectional study, a randomized design that matches participants by age and BMI would offer more robust and generalizable results. Larger prospective trials, ideally randomized and controlled, are needed to establish whether dual priming agents can reliably reduce the induction-to-delivery interval in nulliparous women under a variety of clinical conditions.

## Conclusions

In this study, we compared two dual-priming methods for labor induction in nulliparous women. Overall, there was no statistically significant difference in the induction-to-delivery interval between the two groups, although the Foley catheter with concomitant oxytocin (Pitocin) group showed a marginal two-hour advantage. Notably, the normal vaginal delivery rate was significantly higher in the Foley with Pitocin group.

Cesarean delivery rates and both maternal and neonatal outcomes were comparable across the two methods, indicating no clear benefit or drawback for either approach. However, labor induction rates have substantially increased over recent decades and are likely to continue rising due to escalating maternal morbidities. Given these factors, combination methods of induction should be encouraged to shorten labor without increasing morbidity for mothers and newborns. Future research with larger sample sizes and randomized designs - including broader patient populations - will help clarify optimal strategies for dual-priming labor induction.
